# Phytanic Acid Intake and Lifestyle Modifications on Quality of Life in Individuals with Adult Refsum Disease: A Retrospective Survey Analysis

**DOI:** 10.3390/nu15112551

**Published:** 2023-05-30

**Authors:** Jeffrey J. Li, Jane J. Kim, Fauzia Nausheen

**Affiliations:** Department of Education, California University of Science and Medicine, Colton, CA 92324, USA

**Keywords:** adult Refsum disease, rare disease, phytanic acid, quality of life, retinitis pigmentosa, PHYH, patient registry, chronic disease, physical function, dietary modification

## Abstract

Adult Refsum disease (ARD) is a rare peroxisomal biogenesis disorder inherited in an autosomal recessive fashion and is often characterized by retinitis pigmentosa, cerebellar ataxia, and polyneuropathy. Many patients with ARD require diet modification, psychosocial support, and various specialist visits to manage their symptoms. In this study, we explored the quality of life in individuals with ARD by analyzing retrospective survey data collected by the Coordination of Rare Diseases at Sanford (CoRDS) Registry and Global Defeat Adult Refsum Everywhere (DARE) Foundation. Statistical tests used were frequencies, mean, and median. There were 32 respondents, ranging between 11 and 32 responses for each question. The mean age at diagnosis was 35.5 ± 14.5 years (range 6–64) with 36.4% male and 63.6% female respondents. The average age for retinitis pigmentosa diagnosis was 22.8 ± 15.7 years (range 2–61). Dieticians were the most frequently seen (41.7%) for management of low-phytanic-acid diets. Most participants exercise at least once per week (92.5%). Depression symptoms were reported in 86.2% of the participants. Early diagnosis of ARD is important for managing symptoms and preventing progression of visual impairment due to phytanic acid buildup. Interdisciplinary approach should be used for patients to address physical and psychosocial impairments of ARD.

## 1. Introduction

**Figure 1:**
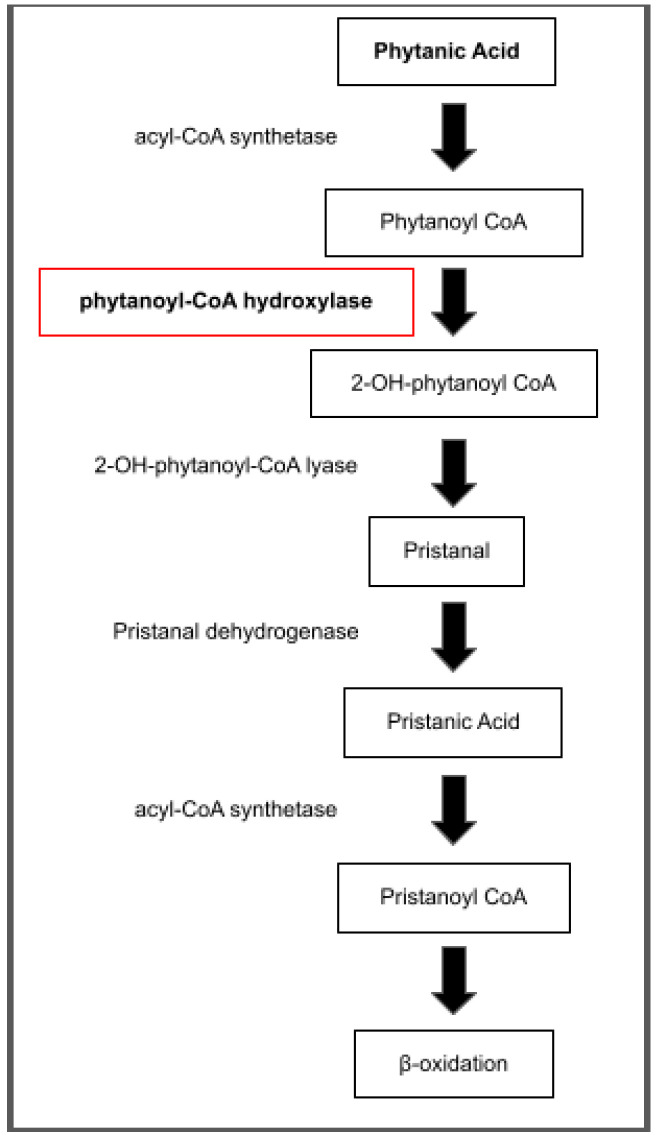
Phytanic acid oxidation pathway. The red box indicates the enzyme deficient with PHYH mutations in adult Refsum disease.

Refsum disease is divided into two subclasses: adult Refsum disease (ARD) and infantile Refsum disease (IRD). Adult Refsum disease, also called heredopathia atactica polyneuritiformis, is a very rare peroxisomal biogenesis disorder inherited in an autosomal recessive fashion and often characterized by retinitis pigmentosa, cerebellar ataxia, polyneuropathy, and increased protein in cerebrospinal fluid (CSF) without an increase in cell count [1]. In ARD, patients have an increase in phytanic acid (3,7,11,15-tetramethylhexadecanoic acid), a saturated branched-chain fatty acid, in their bodies due to a defect in the alpha-oxidation of phytanic acid [2,3]. While the majority of patients with ARD have a deficiency in phytanoyl-CoA hydroxylase (PAHX), which is encoded by the phytanoyl-CoA 2-hydroxylase (PHYH) gene, some patients instead have a deficiency of the type 2 peroxisomal targeting signal (PTS2) receptor encoded by peroxin (PEX)7. The alpha oxidation pathway of phytanic acid is presented in Figure 1.

In IRD, mutations in 12 different genetic loci have been identified as contributing to IRD’s presentation, including peroxisome ATPases PEX1, PEX2, and PEX26. The primary mutation of PAHX implicated in ARD results in increases of primarily phytanic acid, whereas the multiple mutations in IRD lead to increases in very long-chain fatty acids (VLCFA), di- and tri-hydroxycholestanoic acid, pipecolic acid, as well as phytanic acid [4]. While IRD patients typically present in the first year of life, ARD often presents from late childhood to adulthood. However, there have been cases of both IRD patients presenting in adulthood and ARD patients presenting in childhood [5,6]. Both subclasses have similar presentations of visual changes, hearing loss, and cerebellar ataxia. However, patients with IRD can have additional signs of craniofacial dysmorphism, intellectual disability, and gastrointestinal disorders, whereas patients with ARD may present with ichthyosis, kidney malfunction, and bilateral shortening of the metacarpals or metatarsals [7]. There are no official estimates of ARD prevalence. Most cases of ARD in literature reside in the United Kingdom or Norway, where ARD is better known than in other countries. ARD prevalence in the United Kingdom is estimated to be one in 1,000,000 with even lower prevalence in other Caucasian countries, such as the United States [8].

ARD has a wide range of phenotypic presentations, but traditionally presents in late childhood with night vision deterioration, retinitis pigmentosa, and anosmia. The diagnosis for ARD is often made through clinical correlation of symptoms and biallelic mutations in either PHYH or PEX7 [2]. If genetic testing is not diagnostic, then a decrease in activity of phytanoyl-CoA hydroxylase enzyme can also be diagnostic. From 10 to 15 years after diagnosis, symptoms include ataxia, deafness, polyneuropathy, ichthyosis, and cardiac arrhythmias. Bony changes such as shortened metacarpals or metatarsals can also indicate possible ARD. After clinical or molecular diagnosis is made, ARD is normally confirmed with elevated phytanic acid levels (>200 µmol/L) [9]. Phytanic acid causes damage by excessively depositing in various tissues. Food products rich in phytanic acid include meat from ruminant animals, such as beef, lamb, mutton, and goat [2]. Additionally, almonds, coconuts, and peanuts are high in phytanic acid. In terms of treatment, ARD is treated with reduction of phytanic acid via reducing phytanic acid in the diet or eliminating phytanic acid via plasmapheresis or lipid apheresis [10]. Although decreasing plasma phytanic acid may symptomatically treat ARD patients in terms of ichthyosis, sensory neuropathy, and ataxia, decreasing serum phytanic acid may not resolve symptoms of retinitis pigmentosa, deafness, and anosmia [11,12].

Many patients with ARD modify their diet, undergo routine plasma exchange or lipid apheresis, and require accommodations in their daily lives. Some patients prefer regular plasma exchange alongside a low-phytanic-acid diet, whereas others prefer dietary modification only. Although plasmapheresis effectively removes phytanic acid in the short-term, the procedure is expensive and increases the risk of morbidity because of its invasive mechanism. However, diet modifications can be difficult for patients due to strict food restriction, although dietary recommendations for patients with ARD have grown more comprehensive since its inception [13]. Since treatment is not efficacious in reducing retinitis pigmentosa, many patients with ARD suffer from vision loss due to the early onset of visual symptoms in the disease course. The quality of life for patients with ARD is often decreased even with regular treatment and dietary compliance. However, recent advances in stem-cell therapy for retinitis pigmentosa have shown increased retinal neurogenesis and decreased retinal inflammation in mice, indicating a possible clinical treatment for retinitis pigmentosa in the future [14]. Another possible treatment for ARD overall would be chemical co-substrate rescue of PAHX deficiencies due to PHYH mutations, as illustrated in vitro by previous studies [15].

Increasing awareness of ARD can increase early diagnosis of ARD in more patients, as well as support new methods for treatment and symptomatic accommodation. Our study explored the quality of life in individuals with ARD by analyzing retrospective survey data. As one of the first studies to explore global survey data from individuals with ARD, we sought to increase understanding of the clinical and lifestyle trends in individuals with ARD.

## 2. Materials and Methods

We would like to thank the Coordination of Rare Diseases at Sanford (CoRDS) Registry and the Global Defeat Adult Refsum Everywhere (DARE) Foundation for where this data originated. Participants with ARD answered both the “CoRDS Questionnaire” and “Global DARE Foundation Patient Survey Questionnaire”. The “CoRDS Questionnaire” was a 79-question form surveying patient demographics. The “Global DARE Foundation Patient Survey Questionnaire” was a 63-question survey tailored to asking questions specifically about ARD.

The Refsum Patient Registry is the first ever ARD registry and was established by the Global DARE Foundation in August 2020 in partnership with Sanford Research. Submissions were accepted starting July 2020. This study took place from July 2020 to February 2023. The collected data was de-identified by Sanford Research. The data obtained were approved for publication by the CoRDS registry and Global DARE Foundation; Institutional Review Board approval HS-2023-02 was obtained from the California University of Science and Medicine (CUSM). Access to this data can be acquired through approval from the CoRDS Registry website.

The aim of this retrospective survey analysis was to discuss the effects of ARD on quality of life. The requirement was that the participant have a diagnosis of ARD. Participant ages ranged from 7 to 79 years. Legal guardians filled out the forms if the participant was under 18 years. The number of responses to the different questions in these surveys varied because not every question was answered by each individual. Quality of life factors extracted and reported from this survey included demographics, symptoms, details surrounding ARD diagnosis, specialists seen, and medical tests conducted. Also described were the impacts of ARD on physical health, mental health, physical activity, and diet. Statistical tests used were frequencies, mean, and median.

## 3. Results

### 3.1. Demographics

Thirty-two individual participants responded to these self-reported surveys. The number of responses for each question varied between 11 and 32. The mean age was 47.4 ± 17.2 years (range 7–79) at time of survey submission with 36.4% male and 63.6% female respondents (Table 1). The mean age at diagnosis was 35.5 ± 14.5 years (range 6–64) and the mean age of first symptom onset was 18.5 ± 16.8 years (range 1–64). There were nine different countries that participants were born in. The participants’ country of birth with the most responses came from the United States of America (23.8%) and the United Kingdom (23.8%). Responses also came from Austria (14.3%), Australia (9.5%), South Africa (9.5%), Canada (4.8%), Netherlands (4.8%), Ireland (4.8%), and Argentina (4.8%). For employment status, 29.6% were employed full-time, 22.2% were disabled and unable to work, 14.8% were students, 14.8% were retired, 11.1% were employed part-time, 3.7% were stay-at-home parents, and 3.7% were not employed and not looking for work. Of the individuals who were not employed, most (61.1%) were not able to work due to ARD.

### 3.2. Common Symptoms

**Figure 2:**
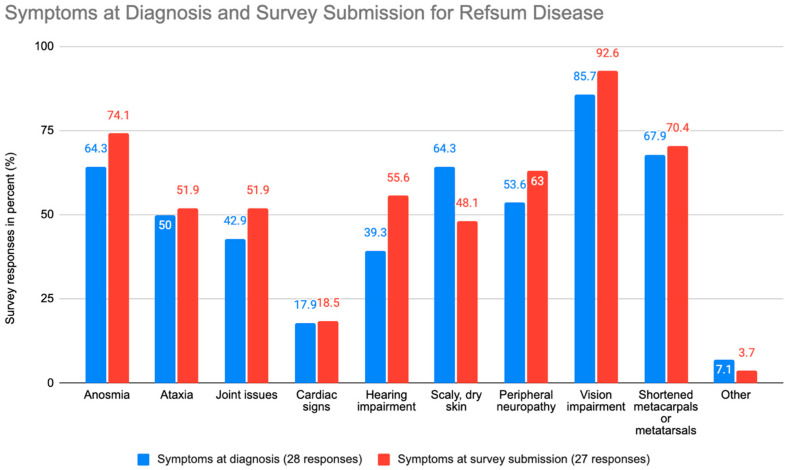
Graph illustrating the frequency of ARD symptoms at diagnosis and survey submission.

The most common symptom at diagnosis of ARD was vision impairment (85.7%), followed by shortened metacarpals or metatarsals (67.9%), dry skin (64.3%), anosmia (64.3%), peripheral neuropathy (53.6%), ataxia (50.0%), joint issues (42.9%), hearing impairment (39.3%), cardiac issues (17.9%), and other (7.1%) (Figure 2). The most common symptom of ARD at survey submission was also vision impairment (92.6%), followed by anosmia (74.1%), shortened metacarpals or metatarsals (70.4%), peripheral neuropathy (63.0%), hearing impairment (55.6%), ataxia (51.9%), joint issues (51.95%), dry skin (48.1%), cardiac issues (18.5%), and other (3.7%).

### 3.3. Retinitis Pigmentosa, Gene Mutations, and Hospitalizations

The mean age of retinitis pigmentosa diagnosis was 22.8 ± 15.7 years (range 2–61) (Table 2). Average phytanic acid levels at ARD diagnosis were 689.1 ± 1188.2 µmol/L (range 16–4243). The most common gene mutation was PHYH (69.6%) and the least common was PEX7 (4.3%). A total of 26.1% of respondents did not know their type of gene mutation. Some participants required visual accommodations, such as signal canes (20.8%), long canes (12.5%), and guide dogs (8.3%). A total of 64.6% received cataract surgery. For ARD-related hospitalizations, 12.0% were hospitalized more than five times, 20.0% were hospitalized fewer than five times, 20.0% were hospitalized only at diagnosis, and 48.0% never had any ARD-related hospitalizations.

### 3.4. Specialists and Medical Tests

**Figure 3:**
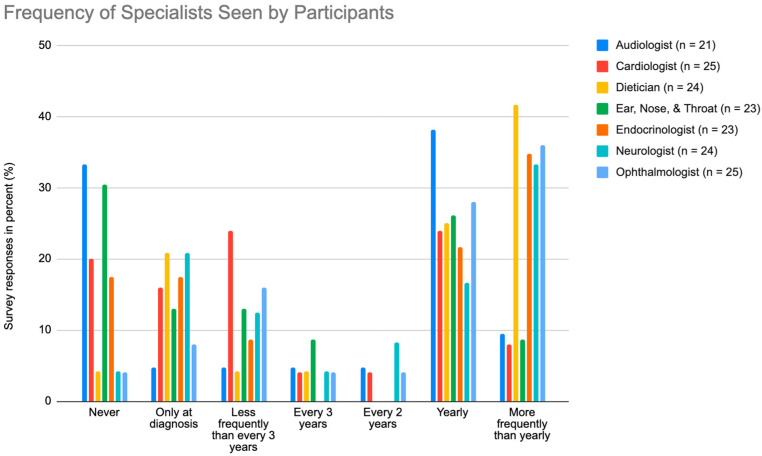
Graph illustrating the frequency participants visit the audiologist, cardiologist, dietician, ENT, endocrinologist, neurologist, and ophthalmologist, where “n” is the number of responses.

As shown in Figure 3, the most common specialist seen “more frequently than yearly” was the dietician (41.7%) and the most common specialist seen “yearly” was the audiologist (38.1%). At “every 2 years”, the neurologist was the most frequently seen (8.3%), and the most frequent for “every three years” was the Ear, Nose, and Throat (ENT) at 8.7%. The most common specialist that participants “never” saw was the audiologist (33.3%).

**Figure 4:**
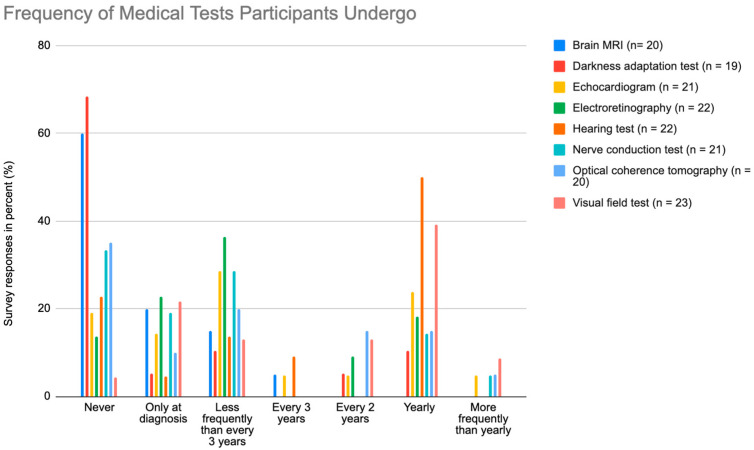
Graph illustrating the frequency participants undergo brain MRI, darkness adaptation testing, echocardiograms, electroretinographies, hearing tests, nerve conduction tests, optical coherence tomographies, and visual field tests, where “n” is the number of responses.

As shown in Figure 4, the medical tests performed most at “more frequently than yearly” was the visual field test (8.7%). The hearing test was the most common test that participants received annually (50.0%). At every two years, the optical coherence tomography was the most frequent test (15.0%), and the most frequent every three years was the hearing test again (9.1%). The least common test was the darkness adaptation test with 68.4% of participants having never received it.

### 3.5. Limitations with Health, Activity, and Pain

A total of 68.9% of the participants rated their general health as “good” or better while 31.0% rated their health as only “fair” or “poor” (Table 3). A total of 34.5% responded that their health “always” limited them from doing vigorous activities. A total of 44.8% stated that their health “often” or “sometimes” limited their ability to partake in vigorous activities. Only 20.7% “rarely” or “never” had their activities limited by their health. The majority also had daily pain levels of moderate (11.5%) and low (42.3%). A total of 46.2% had no daily pain. Pain interfered with the participants’ enjoyment of life always (3.4%), often (20.7%), sometimes (20.7%), rarely (34.5%), and never (20.7%). Most of the participants were discouraged with having ARD with 88.4% being discouraged “a little bit of the time” up to “all of the time”. Only 11.5% did not feel discouraged at all with their diagnosis. Similarly, 88.5% of participants were worried about having ARD “a little bit of the time” up to “all of the time” with 11.5% worried “none of the time”. When asked how often the participants felt depressed, 6.9% responded “always”, 6.9% answered “often”, 20.7% reported “sometimes”, 51.7% answered “rarely”, and 13.8% replied “never”.

### 3.6. Mobility and Exercise

According to Table 4, 59.3% of participants had high mobility and 40.7% had impaired mobility categorized as “moderate” mobility, “limited mobility”, or “no mobility”. When asked about their level of balance, 40.7% had “good” balance, 44.4% had “moderate” balance, and 14.8% had “poor” balance. The majority of participants also exercise at least once per week (92.5%). A total of 3.7% exercise once per week, 33.3% exercise two to three days per week, 33.3% exercise four to six days per week, and 22.2% exercise the full seven days per week. Participants were also asked how many minutes per week they exercise. The majority (42.3%) exercised more than 150 min per week. When asked about the intensity of the exercises, the responses were “no exercise” (3.8%), “light” exercise (57.7%), “moderate” exercise (26.9%), and “hard” exercise (11.5%). A total of 52% reported that ARD affected their ability to perform certain exercises.

### 3.7. Modifications in Diet

The frequency of phytanic acid level measurement and effects of ARD on diet were discussed in Table 5. Phytanic acid levels were measured “more often than every month” (3.8%), “every month” (7.7%), “every other month” (3.8%), “every 3 months” (11.5%), “every 6 months” (23.1%), “yearly” (26.9%), “never” (3.8%), and “unknown” (19.2%). A total of 96.2% were currently on a low-phytanic-acid diet; one participant (3.8%) was currently not on a low-phytanic-acid diet. A total of 60.0% were “very strict” with their compliance of their low-phytanic-acid diet, 36.0% were “good” with their compliance, and 4.0% (one response) were “intermittent” in terms of following the diet. A total of 21.7% snack fewer than three times a day, 73.9% snack three to six times a day, and 4.3% snack more than six times per day. The longest periods where individuals did not eat anything were less than two hours (8.7%), two to three hours (26.1%), four to six hours (56.5%), and greater than six hours (8.7%). The impacts of low-phytanic-acid diets on participants’ quality of life have been high (13.0%), moderate (26.1%), low (47.8%), and none (13.0%).

## 4. Discussion

This study provides an analysis of the general lifestyle of individuals with ARD, including the psychological, physical, and social toll ARD played in their daily lives. We used the data from the Global Patient Registry for Refsum Disease, a database associated with the Global DARE Foundation.

Although ARD has heavy clinical implications for patients’ daily lives, it is rarely diagnosed and treated at onset. The first onset of symptoms until medical diagnosis of ARD in the survey group had an average gap of 17 years, demonstrating the need for increased awareness of ARD and its early diagnosis. The youngest survey respondent was 6 years old, supporting the notion that ARDS can be diagnosed in childhood and therefore treated earlier due to the disease’s mechanism through inherited gene mutations [6]. In addition, symptoms such as retinitis pigmentosa were diagnosed at an early age (22.8 years). However, despite diagnosing retinitis pigmentosa early, the underlying cause of ARD was rarely identified, and visual symptoms worsened with 41.7% of participants requiring a cane or guide dog because of their deteriorated vision. As one of the most prevalent symptoms with severe consequences towards daily life, patients with retinitis pigmentosa may benefit from early testing for ARD to expedite treatment and prevent progression of vision loss.

Within the questionnaire answers, participants responded to the survey at an average of 12 years after ARD diagnosis. At the time of ARD diagnosis, 85.7% of participants had vision impairment and 64.3% had dry skin. By the time of survey submission, vision impairment increased to 92.6% of participants, and dry skin decreased to 48.1% of participants. The percentage of individuals who acquired hearing loss increased by over 15% from time of diagnosis to time of survey submission. These data support previous research that stated a low-phytanic-acid diet is effective towards improving dry skin but relatively ineffective in improving vision impairment, deafness, or anosmia [11]. Therefore, early diagnosis is vital to prevent symptoms that are irreversible with the current available treatment regimens.

In total, 69.6% of participants reported their known gene mutation was PHYH, despite previous literature stating PHYH gene mutations were the cause of ARD in greater than 90% of ARD patients [16]. The likely explanation for this discrepancy was that 26.1% of participants did not know their ARD-related gene mutation. Patients who did not understand the details of their own condition may feel a lack of control and motivation over their condition and outcomes; promoting understanding in patients with chronic conditions can help improve feelings of autonomy as well as treatment adherence [17].

Due to the wide range of severe clinical manifestations of ARD, a fifth (22.2%) of the participants were disabled and not able to work, and many participants required visiting more than one specialist regularly to manage symptoms and screen for new possible physical deterioration. The most frequently visited specialist was the dietician, with 41.7% patients visiting regularly. Since one of the hallmarks of ARD treatment is dietary modifications to lower phytanic acid intake, regular dietician visits likely increase compliance and provide necessary dietary adjustments and phytanic acid level monitoring. Following dietician visits, ophthalmologist visits were the next most frequent specialist visits (36.0%) for ARD patients. As of now, vision impairment is not effectively treated with current therapy and was the most common symptom (92.6%) among survey respondents [18]. In addition, visual impairment affected the daily lives of ARD patients in mobility and work more than symptoms such as anosmia or bony malformations, increasing the likelihood of compliance with vision-related specialist visits. Along a similar vein, the most common screening among ARD patients were yearly hearing tests, likely due to the progression of deafness caused by ARD and its burden on daily life.

The majority of participants dealt with daily pain and suffered from depressive symptoms. In this study, 53.8% of respondents had everyday “low” or “moderate” amounts of pain, and 40.7% had their mobility affected by the disease. In addition, depression symptoms were reported in 86.2% of the participants, aligning with the high rates of depression in individuals with other chronic conditions, such as COPD, heart disease, and multiple sclerosis [19,20,21]. As a chronic condition with no definitive cure, ARD patients would likely benefit from psychosocial support from regular therapy sessions with specialists, as well as an interdisciplinary approach to help alleviate the various stresses of ARD.

Many participants shifted their daily habits in terms of diet and exercise to manage their physical changes in pain, mobility, and mood. Dietary modifications for ARD patients included not only low phytanic acid intake, but also preventing breakdown of adipose tissue by avoiding a fasting state with frequent meals or snacks [2]. Breakdown of adipose tissue releases stored phytanic acid into the plasma. Most respondents (91.3%) avoided fasting and did not go without meals or snacking for longer than six hours. This most likely helped decrease the chance of releasing more phytanic acid into the blood, reducing exacerbation of ARD-related symptoms. In addition, 42.3% of individuals with ARD exercised more than 150 min a week. Exercise was likely beneficial in maintaining the mobility, muscle strength, and cardiovascular health compromised by ARD. Previous research had shown that exercise improved depressive symptoms in other chronic conditions and was observed to be one of the most helpful adjuvant treatments for depression, with yoga and mindfulness meditation providing lower but comparable benefits as well [21,22].

This study has some limitations. The first limitation of this study was its reliance on self-reported answers. Individual perceptions of symptoms, diet compliance, specialist visits, exercise routines, and physical and mental well-being guided our study and its findings. A study built on observable, measurable variables would likely aid efforts towards creating more objective depictions of ARD clinical trends. For example, monitoring changes in phytanic acid levels after exercising could reveal a possible threshold between the benefits of improving cardiovascular health versus worsening the stress and energy needs of individuals with ARD. The second limitation was the low number of responses. Since 2021, there have been over 200 confirmed cases of ARD [2], but there were only 32 respondents to the ARD questionnaire. This could be explained by a multitude of reasons. Firstly, the Global Patient Registry for Refsum Disease was only created within the past three years. Secondly, the ARD questionnaire is currently only offered in English. Thirdly, many clinicians and patients may not be actively thinking about ARD as a differential due to its rarity in diagnosis. By continuing the existence of this registry for the long-term future, offering the survey in different languages, and raising awareness of ARD, we may be able to increase the number of respondents to further scientific research into ARD. The third limitation was the time-point that these responses were collected. Instead of surveying patients at seemingly random points after their ARD diagnosis, it would be more insightful to be able to follow at symptom- or diagnosis-onset and observe trends at more discrete levels. However, we acknowledge this was most likely not possible because of how recently the Global Patient Registry for Refsum Disease was created and the overall difficulty of following patients long-term. Future research could follow ARD patients over the period of their lives to document the needs of those who have life-altering ARD symptoms.

## 5. Conclusions

The CoRDS Registry collects useful information on the quality-of-life factors of individuals with ARD. This manuscript will hopefully serve to increase awareness of ARD and the general clinical manifestations and lifestyles of patients with ARD. If readers of this manuscript have or know someone who has a medical diagnosis of ARD, then we encourage them to register with the Global Patient Registry for Refsum Disease to help further research into the rare disease that is Adult Refsum Disease [23].

## Figures and Tables

**Table 1:** Demographics of survey responses.

Age at survey submission, years			
n = 32			
Mean	Standard deviation (SD)	47.4	17.2
Median	min, max	48	7, 79
Age at diagnosis, years			
n = 24			
Mean	SD	35.5	14.5
Median	min, max	37	6, 64
Age at first symptoms, years			
n = 15			
Mean	SD	18.5	16.8
Median	min, max	15	1, 61
Race			
n = 28		n	%
White		26	92.9
Asian		1	3.6
Other/Refuse to answer		1	3.6
Sex at birth			
n = 22		n	%
Male		8	36.4
Female		14	63.6
Country of birth			
n = 21		n	%
United States of America		5	23.8
Australia		2	9.5
Canada		1	4.8
UK		5	23.8
Netherlands		1	4.8
South Africa		2	9.5
Ireland		1	4.8
Argentina		1	4.8
Austria		3	14.3
Which of the following categories best describes the participant’s employment status?			
27 responses		n	%
Student		4	14.8
Employed, part-time		3	11.1
Employed, full-time		8	29.6
Stay at home parent		1	3.7
Not employed, looking for work		0	0
Not employed, not looking for work		1	3.7
Retired		4	14.8
Disabled, not able to work		6	22.2
If not working or working part-time, is this due to Refsum disease?			
n = 18		n	%
Yes		11	61.1
No		7	38.9

Parameters reported were age at survey submission, diagnosis, and first symptoms. Other collected data were race, sex at birth, country of birth, and employment status.

**Table 2:** Details about adult Refsum disease diagnosis.

Age at retinitis pigmentosa diagnosis, years			
n = 23			
Mean	Standard deviation (SD)	22.8	15.7
Median	min, max	18	2, 61
Phytanic acid levels at diagnosis, µmol/L			
n = 11			
Mean	Standard deviation (SD)	689.1	1188.2
Median	min, max	375	16, 4243
Gene mutation			
n = 23		n	%
PHYH		16	69.6
PEX7		1	4.3
Unknown		6	26.1
Participant received cataract surgery			
n = 25		n	%
Yes		16	64.0
No		9	36.0
Participant’s vision required use of the following:			
n = 24		n	%
Guide dog		2	8.3
Long cane		3	12.5
Signal cane		5	20.8
None		14	58.3
Refsum-related hospitalizations			
n = 25		n	%
None		12	48.0
Only at diagnosis		5	20.0
<5 since diagnosis		5	20.0
>5 since diagnosis		3	12.0

Additional information surrounding the participants’ ARD diagnosis. Reported parameters included age of retinitis pigmentosa diagnosis, phytanic acid levels at diagnosis, gene mutation involved, any cataract surgery, accommodations required vision loss, and hospitalizations related to ARD.

**Table 3:** Impact of Refsum disease on daily physical and mental health.

In general, would the participant say his/her health is…		
n = 29	n	%
Poor	4	13.8
Fair	5	17.2
Good	13	44.8
Very good	6	20.7
Excellent	1	3.4
Does the participant’s health currently limit him/her in doing vigorous activities?		
n = 29	n	%
Always	10	34.5
Often	5	17.2
Sometimes	8	27.6
Rarely	4	13.8
Never	2	6.9
What is the participant’s typical daily pain level?		
n = 26	n	%
None	12	46.2
Low (1 to 3)	11	42.3
Moderate (4 to 6)	3	11.5
High (7 to 10)	0	0
How much does pain interfere with the participant’s enjoyment of life?		
n = 29	n	%
Always	1	3.4
Often	6	20.7
Sometimes	6	20.7
Rarely	10	34.5
Never	6	20.7
Is the participant discouraged about having Refsum disease?		
n = 26	n	%
None of the time	3	11.5
A little bit of the time	7	26.9
Some of the time	10	38.5
A good bit of the time	4	15.4
Most of the time	1	3.8
All of the time	1	3.8
Is the participant worried about having Refsum disease?		
n = 26	n	%
None of the time	3	11.5
A little bit of the time	6	23.1
Some of the time	12	46.2
A good bit of the time	2	7.7
Most of the time	0	0
All of the time	3	11.5
How often does the participant feel depressed?		
n = 29	n	%
Always	2	6.9
Often	2	6.9
Sometimes	6	20.7
Rarely	15	51.7
Never	4	13.8

Perceptions about the participants’ own health, ability to participate in activities, pain level, effects on enjoyment of daily life, and overall mental well-being.

**Table 4:** Impact of Refsum disease on physical activity.

What is the participant’s level of mobility?		
n = 27	n	%
No mobility	1	3.7
Limited (requires full assistance)	2	7.4
Moderate (walk semi-assisted, need a railing to walk up and down stairs)	8	29.6
High (can walk unassisted, very steady, able to walk on uneven surfaces)	16	59.3
How would the participant describe the current level of balance?		
n = 27	n	%
Good	11	40.7
Moderate	12	44.4
Poor	4	14.8
How many days in a week does the participant usually exercise?		
n = 27	n	%
0 days	2	7.4
1 day	1	3.7
2–3 days	9	33.3
4–6 days	9	33.3
7 days	6	22.2
Over the course of one week, what is the total number of minutes the participant exercise or does an activity?		
n = 26	n	%
Not active/does not exercise	1	3.8
<30 min	0	0
31–60 min	4	15.4
61–90 min	7	26.9
91–120 min	2	7.7
121–150 min	1	3.8
>150 min	11	42.3
What is the participant’s maximum exercise intensity level?		
n = 26	n	%
No exercise	1	3.8
Light (yoga, walking)	15	57.7
Moderate (cycling, swimming)	7	26.9
Hard (running, interval training)	3	11.5
Has Refsum disease prevented the participant from doing certain type(s) of exercise?		
n = 25	n	%
Yes	13	52.0
No	12	48.0

The parameters measured were the participants’ mobility, level of balance, amount of exercise, level of exercise, and limitations on exercise.

**Table 5:** Effects of Refsum disease on diet.

How often does the participant have a Phytanic Acid Level done?		
n = 26	n	%
More often than every month	1	3.8
Every month	2	7.7
Every other month	1	3.8
Every 3 months	3	11.5
Every 6 months	6	23.1
Yearly	7	26.9
Never	1	3.8
Unknown	5	19.2
Is the participant on a low-phytanic-acid diet?		
n = 26	n	%
Yes	25	96.2
No	1	3.8
What is the participant’s compliance with the low-phytanic-acid diet within the last year?		
n = 25	n	%
Very strict	15	60
Good	9	36
Intermittent	1	4
Poor	0	0
Not following the diet	0	0
How many times a day does the participant eat, including snacks?		
n = 23	n	%
<3	5	21.7
3–6	17	73.9
>6	1	4.3
On average, what is the longest period during the day the participant would go without eating between meals/snacks?		
n = 23	n	%
<2 h	2	8.7
2–3 h	6	26.1
4–6 h	13	56.5
>6 h	2	8.7
What impact does the low-phytanic-acid diet have on the participant’s quality of life? (e.g., eating out, social activities)		
n = 23	n	%
No impact	3	13
Low impact	11	47.8
Moderate impact	6	26.1
High impact	3	13

Table shows the frequency of phytanic acid levels and the effects of having adult Refsum disease on diet.

## Data Availability

The data that support the findings of this study are available from Coordination of Rare Diseases at Sanford (CoRDS), but restrictions apply to the availability of these data, which were used under license for the current study, and so are not publicly available. Data are, however, available from CoRDS upon reasonable request and approval.
